# Single-cell transcriptomics reveals comprehensive microenvironment and highlights the dysfuntional state of NK cells in endometrioid carcinoma

**DOI:** 10.1097/MD.0000000000037555

**Published:** 2024-03-29

**Authors:** Wenjie Shi, Wuchen Wu, Jing Wang, Xianghong Meng

**Affiliations:** a Department of Medical Technology, Beijing Health Vocational College, Beijing, China; b Neurosurgery Department of Shenzhen University General Hospital, Shenzhen, China.

**Keywords:** endometrioid endometrial cancer, LAMP3+ dendritic cells, natural killer cell, tumor microenvironment, tumor-associated macrophages

## Abstract

Endometrioid endometrial cancer (EEC) is one of the most common gynecologic malignancies. The interaction between cancer cells and the cells in the tumor microenvironment (TME) plays a crucial role in determining disease progression and response to treatment. To better understand the diversity in the TME of ECC, we conducted a comprehensive analysis using single-cell RNA sequencing across 21 samples, including 16 ECC and 5 adjacent normal tissues. We primarily focused on tumor-infiltrating natural killer (NK) cells and their cell–cell interactions with other immune cell types. We identified a CD56dim_DNAJB1 NK cells subset, which had low cytotoxic capability and high stress levels, suggesting a dysfunctional state. This subset showed strong interactions with tumor-associated macrophages through several ligand–receptor pairs. Additionally, we observed that tumor-infiltrating LAMP3+ dendritic cells may inhibit CD8+ T cells or attract regulatory T cells to the tumor area. These dendritic cells also had impaired activation effects on NK cells within the TME. Our study provides valuable insights into the role of NK cells in cancer immunity and highlights the potential of targeting specific NK cell subsets for therapeutic purposes.

## 1. Introduction

Endometrial cancer (EC) is a common gynecologic malignancy that is increasing in prevalence worldwide.^[1]^ The primary histological subtype of EC is endometrioid carcinoma (ECC), which accounts for approximately 65% to 85% of all EC cases.^[2]^ While most ECC patients can be diagnosed at an early stage and effectively treated with hysterectomy, it is important to consider that this surgical approach may not be suitable for all cases, particularly those involving very young or elderly patients.^[3]^ Therefore, there is an urgent need to explore and develop alternative treatment modalities. Immunotherapies, such as chimeric antigen receptor (CAR)-T cell and CAR-NK cell therapies, have recently emerged as promising additions to the traditional treatment options for various types of cancer.^[4,5]^ However, it is crucial to acknowledge that tumors are complex ecosystems with extensive heterogeneity, which significantly influences tumor progression and treatment outcomes.^[6,7]^ Therefore, a meticulous delineation of the cellular constituents becomes imperative in this context.

The tumor microenvironment (TME) is composed of malignant cells, immune cells, and stromal cells, and it plays a crucial role in tumorigenesis, prognosis, and metastasis.^[6]^ However, understanding the complex interactions within the TME poses significant challenges, but also provides opportunities for advancements in cancer diagnosis and treatment. Single-cell RNA (scRNA) sequencing has emerged as a powerful tool for identifying different cell types at the individual cell level.^[8]^ By analyzing scRNA data, researchers have observed an increased presence of exhausted CD8+ T cells and tumor-associated macrophages within the cancerous environment.^[9]^ It is important to note that endometrial epithelial cells, specifically the unciliated glandular epithelium, are proved to be the source of endometrioid endometrial cancer (EEC), rather than stromal cells.^[10]^ While previous studies have contributed to our understanding of the TME in EEC, the complex interplay between the TME and its impact on prognosis and resistance to therapy in EEC is still not fully understood.

Natural killer (NK) cells play a crucial role in the TME, contributing to various aspects of tumor regulation, including direct cellular eradication and the secretion of proinflammatory cytokines.^[11]^ In humans, NK cells are primarily classified into 2 groups based on the expression levels of CD56 (NCAM1) and CD16 (FCGR3A): CD56^dim^CD16^hi^ and CD56^bright^CD16^lo^.^[12,13]^ Unlike CD8+ T cells, NK cells serve as alternative reservoirs of cytotoxic activity, providing defense against tumor cells with low mutation burdens and aberrant expression of major histocompatibility complex (MHC) class I molecules.^[14]^ However, the functionality of NK cells can be compromised by the immunosuppressive factors present in the TME. While the dysfunctional state of CD8+ T cells, characterized by diminished cytotoxic potential and increased expression of inhibitory receptors, has been extensively studied,^[15]^ the specifics of NK cell dysfunction remain largely unexplored. Therefore, our study aims to comprehensively examine NK cells, elucidate their heterogeneity, and shed light on the nature of their dysfunctional manifestation in ECC.

In this study, we have collected a large dataset of published scRNA data on ECC, allowing us to thoroughly investigate the heterogeneity of the TME. To our knowledge, our research has compiled the largest collection to date, consisting of 151,502 individual cells from 21 samples. Through our analysis, we have identified state transitions of tumor-infiltrating NK cells, revealing their compromised cytotoxic capabilities. Additionally, we have shed light on the components within the TME that potential have cell–cell communication networks. This dataset serves as a valuable resource for enhancing our understanding of the immune microenvironment. It also offers crucial insights that could potentially guide the development of NK-cell based immunotherapies, opening up new possibilities for more effective cancer treatment strategies.

## 2. Methods

### 2.1. Data collection

In this study, we obtained scRNA datasets of ECC from 4 previously published studies. These datasets were acquired either in original sequencing FASTQ data or the expression matrices. Specifically, we collected the expression matrices of 5 tumor samples from Regner et al (2021)^[16]^ via GSE173682, and 2 tumor samples from^[17]^ Cassier et al (2023) through GSE225691. In our text, we refer to these datasets as “MolC_” and “Nature_” respectively. Additionally, we downloaded original sequencing data from Guo et al (2021)^[9]^ via PRJNA650549, which included 5 tumor samples and 3 normal samples. This dataset is labeled as “Aging_” in our study. Similarly, we obtained sequencing data from Ren et al (2022)^[10]^ via SRP349751, which consisted of 3 tumor samples and 2 normal samples. This dataset is denoted as ‘NC_’ in our analysis. The clinical information were detailed in Table S1, Supplemental Digital Content, http://links.lww.com/MD/L965.

The raw sequencing reads were processed using the Cell Ranger Software Suite (10x Genomics, Cell Ranger 7.2.0), with refdata-gex-GRCh38-2020-A serving as the reference for mapping reads on the human genome (GRCh38/hg38). This process generated the unique molecular identifier (UMI) matrices. In total, our dataset comprised 21 samples, including 16 tumor and 5 normal tissues. It is important to note that the clinical data of patients involved in this study was sourced from open-access databases, rendering an ethical declaration inapplicable. Patients in these databases have obtained ethical approval, and all information about ethical approval of these open-access databases can be obtained from the respective published studies.

### 2.2. Quality control and doublet exclusion

The count matrix was imported into Seurat (version 4.2)^[18]^ using the “Read10X” function for quality control. Initially, genes expressed in fewer than 10 cells were removed to ensure data accuracy. Subsequently, cells with fewer than 500 detected UMI counts or with more than 20% mitochondrial UMI counts were filtered out to eliminate potential outliers. Cells with over 8000 genes were also excluded to prevent potential doublets. In addition to these measures, we utilized the DoubletFinder (version 2.0)^[19]^ tool to identify and exclude potential doublets, thereby improving the reliability of the data. After implementing these rigorous quality control measures, we obtained a total of 151,502 high-quality cells for further analysis.

### 2.3. Integration, clustering, and annotation

Sequentially, all operations were performed in the Seurat software with default parameters, unless otherwise specified. The “FindVariableFeatures” function was used to identify the top 3000 genes with high variability. To address batch effects among samples, we applied the “RunHarmony” function from the Harmony package (Version 1.0),^[20]^ ensuring comparability of our data across different batches. Then we utilized the “FindClusters” function with the top 40 principal components at a resolution of 0.8 to identify distinct cell clusters within our dataset. Finally, cell types were determined using the SingleR package (Version 1.4.1),^[21]^ the CellMarker dataset,^[22]^ and markers extracted from published papers.

### 2.4. Gene set variation analysis

Pathway analyses were conducted to assess the activation of hallmark pathways, as described in the molecular signature database (https://www.gsea-msigdb.org/gsea/msigdb). Then, we applied Gene set variation analysis (GSVA) in the GSVA package (version 1.26.0)^[23]^ to assign pathway activity estimates to individual cells.

### 2.5. Calculation of signature score

We utilized the R package AUCell^[24]^ to compute the signature score for a specific set of genes. First, we constructed a ranked expression matrix using the “AUCell_buildRankings” function. Then, we calculated the Area Under the Curve (AUC) value using the “AUCell_calcAUC” function. To evaluate the functional differences in CD8 T cells between normal and tumor tissues, we defined cytotoxicity and exhaustion gene sets based on a comprehensive review of previous studies.

### 2.6. Definition of cytotoxicity, inflammatory, and stress gene sets

To evaluate the functional differences among NK cell subsets, we created an extensive compilation of cytotoxicity, inflammatory, and stress-related gene sets, as well activating and inhibitory receptors from previous research. The gene sets mentioned in Zhang et al’s paper (refer to Table S2, Supplemental Digital Content, http://links.lww.com/MD/L966 for more information) were included. We computed the scores for each gene set using the “AddModuleScore” function in Seurat.

### 2.7. Receptor ligand interaction analysis

To understand the communications between NK cells and other immune cells, we utilized Cellchat^[25]^ to calculate the ligand -receptor communication networks. CellChat is a computational tool designed to quantitatively infer and analyze cell–cell communication networks from scRNA data, enabling the prediction of major signaling interactions and their coordination in cellular functions. It achieves this by integrating prior knowledge of the interactions among signaling ligands, receptors, and their cofactors.

## 3. Results

### 3.1. Single-cell expression atlas and cell type annotation in the TME of ECC

**Figure 1.**
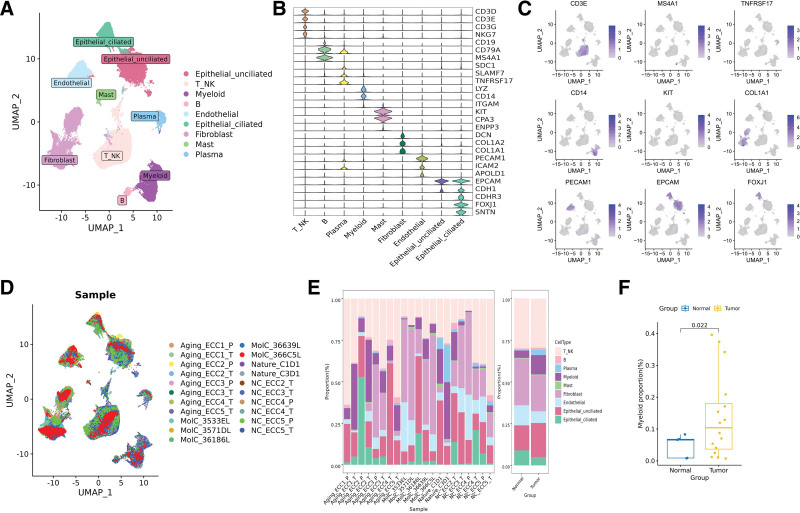
ECC single-cell atlas. (A) UMAP plot of 151,502 individual cells from 21 samples, colored by their 9 major cell types. (B) Violin plot show expression level of markers highly expressed in cell types. (C) Feature plot show log-normalized expression of markers of cell types. (D) UMAP plot of all cells, colored by patients. (E) Major cell type composition of each patient and tumor/normal group. (F) Pairwise *t*-test comparisons revealing the significant enrichment of myeloid cells in tumor.

To investigate the cellular compositions in ECC, we conducted scRNA-seq analysis using tumor sections from 16 patients and adjacent normal tissue sections (n = 5). After stringent quality control and removal of potential doublets, a total of 151,502 high-quality cells were included for further analysis. Among these cells, 119,441 originated from tumors, while 32,061 were from adjacent normal tissues. To address the batch effect, we integrated the scRNA-seq data from different samples using the Harmony algorithm. Using well-established cell markers, we identified 9 main known cell types (Fig. 1A), including T_NK cells (marked by T markers CD3E and CD3G, as well as NK markers NCAM1 and KLRF1), B cells (CD79A and MS4A1), plasma cells (SDC1 and MZB1), myeloid cells (LYZ and CD14), endothelial cells (PECAM1 and VWF), fibroblast cells (DCN and COL1A1), mast cells (KIT and CPA3), and epithelial cells (EPCAM and CDH1) (Fig. 1B). Additionally, we observed 2 distinct cell types within the epithelium: the ciliated epithelium (EPCAM+ CDHR3+ FOXJ1+) and the unciliated epithelium (EPCAM+ CDHR3− FOXJ1−) (Fig. 1B and C), consistent with a recent study^[10]^ that also identified these 2 cell types and suggested their potential functional differences. While all 9 major cell types were identified in both the tumor and adjacent normal tissues, the degree of infiltration for each of these cell types varied, potentially indicating the heterogeneity among ECC patients (Fig. 1D and E). Notably, myeloid cells were significantly more prevalent in tumor group, underscoring their crucial role in ECC progression (Fig. 1F).

### 3.2. Detailed reclustering of T and NK cells

**Figure 2.**
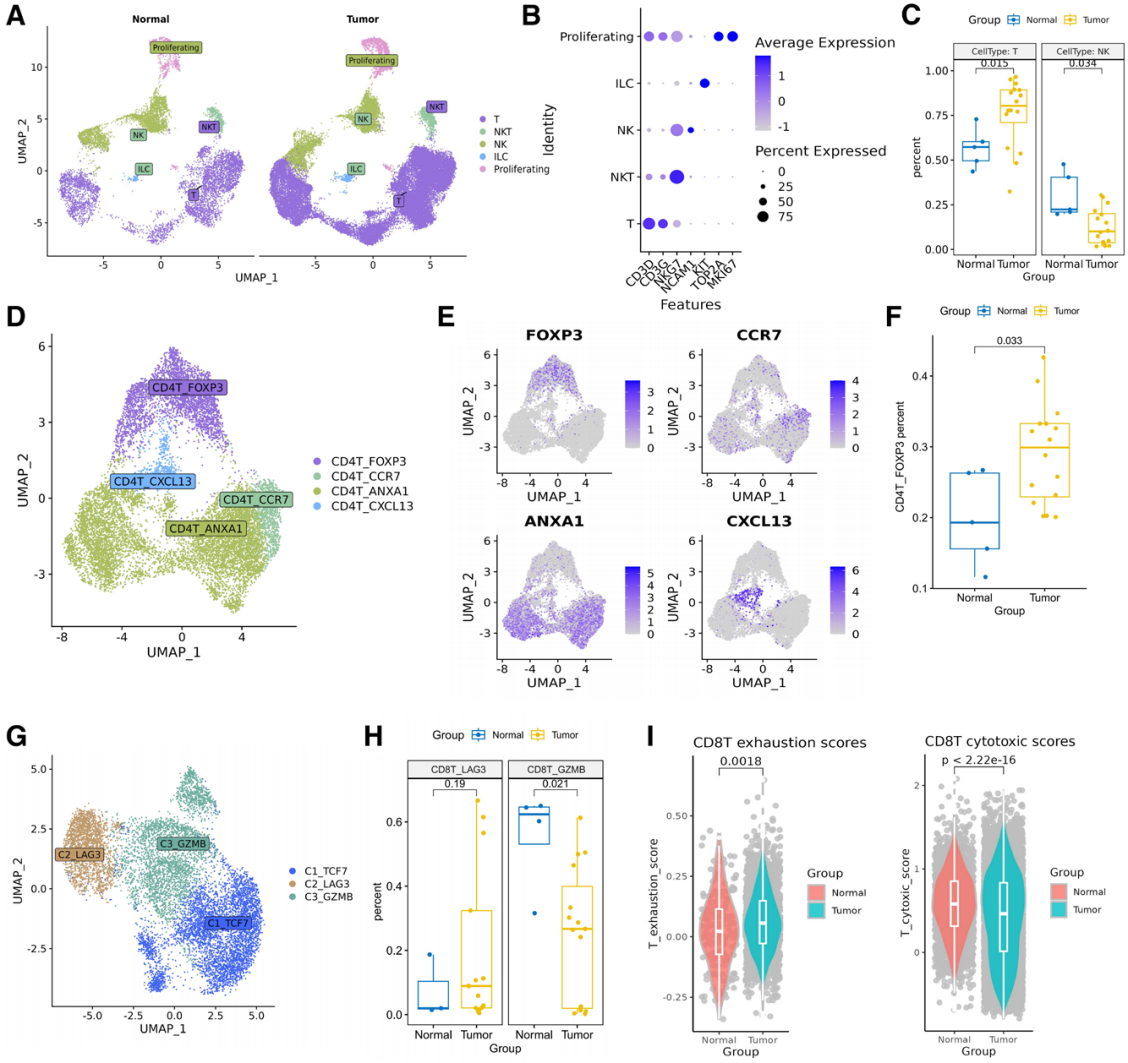
Cellular composition of T cells and subsets of CD4+ and CD8+ T cells. (A) UMAP plot of T_NK cells, colored by their 5 cell types, and split by tumor and normal. (B) Dot plot shows the expression level of markers of T and NK cells. (C) Pairwise *t*-test comparisons revealing the significant enrichment of T cells in tumor and NK cells in normal. (D) UMAP plot of CD4+ T cells, colored by their 4 subsets. (E) Feature plot show log-normalized expression of markers of CD4+ T subsets. (F) Pairwise *t*-test comparisons revealing the significant enrichment of CD4T_FOXP3 cells in tumor. (G) UMAP plot of CD8+ T cells, colored by their 3 subsets. (H) Pairwise *t*-test comparisons revealing the significant enrichment of CD8T_LAG3 cells in tumor and CD8T_GZMB cells in normal. (I) Exhaustion and cytotoxic scores of CD8+ T cell in tumor and normal groups.

T and NK cells have long been proposed as key immune cell types involved in regulating tumorigenesis and cancer progression. However, the precise identity of these heterogeneous cell populations remains unclear in ECC. In this study, we categorized the T/NK cells into 5 distinct types based on specific cellular signature markers previously reported (Fig. 2A). These types included T cells (n = 29,451), identified by the expression of CD3D and CD3G; NK cells (n = 7724), characterized by the expression of NCAM1 (CD56), FCGR3A (CD16) and KLRF1; NKT cells (n = 1287), which expressed both T and NK markers; ILC cells (n = 441), marked by KIT; and Proliferating cells (n = 2234), marked by TOP2A and MKI67 (Fig. 2B). Notably, we found that T cells (*P* = .015) were significantly enriched in tumor tissue compared to adjacent normal tissue, while NK cells (*P* = .035) were more prevalent in normal tissue (Fig. 2C). These findings suggest a complex interplay of immune responses within the TME.

### 3.3. Detailed reclustering of CD4+ T and CD8+ T cells

For the analysis of T cells, we performed 2 rounds of unsupervised clustering. The first round aimed to distinguish between CD4+ T (CD4T) and CD8+ T (CD8T) cells based on the expression of CD4 and CD8A. In the second round, we further divided CD4+ T cells into 4 subsets. CD4T_FOXP3 cells, characterized by high expression of FOXP3, CTLA4, and TIGHT, were identified as regulatory T (Treg) cells. CD4T_CCR7 cells, which exhibited high expression of CCR7 and SELL, were classified as Naive T cells. CD4T_ANXA1 cells showed high expression of ANXA1, a key marker of central memory T cells. CD4T_CXCL13 cells were characterized by the expression of CXCL13 (Fig. 2D and E). As expected, Treg (CD4T_FOXP3) cells were significantly more abundant in tumor tissue compared to adjacent normal tissue (Fig. 2F).

The CD8+ T cells were further divided into 3 subsets (Fig. 2G). CD8T_LAG3 cells, characterized by high expression of LAG3, PDCD1, and TIGHT, were identified as exhausted T (Trex) cells. Naive T cells were classified as CD8T_TCF7 cells, which were characterized by high expression of CCR7 and TCF7. Effector T cells, characterized by high expression of GZMB, GZMA, and PRF1, were classified as CD8T_GZMB cells. Interestingly, CD8T_GZMB cells were significantly reduced in tumor tissue compared to adjacent normal tissue, while CD8T_LAG3 cells were more prevalent in tumor-adjacent normal tissue, although not significantly (Fig. 2H).

The AUCell R package was used to calculate the exhaustion and cytotoxic values for the CD8T_GZMB and CD8T_LAG3 subsets. Compared to normal tissues, tumor-infiltrating CD8T_LAG3 cells had a significantly higher exhaustion score (*P* = .0018), and the effector CD8T_GZMB subset showed statistically reduced cytotoxicity scores (Fig. 2I). Overall, the T cells infiltrating the tumor displayed a dysfunctional state, highlighting the complex interplay of immune responses in the TME.

### 3.4. Detailed analysis of NK cells uncovering CD56dim_DNAJB1 dysfunctional state

**Figure 3.**
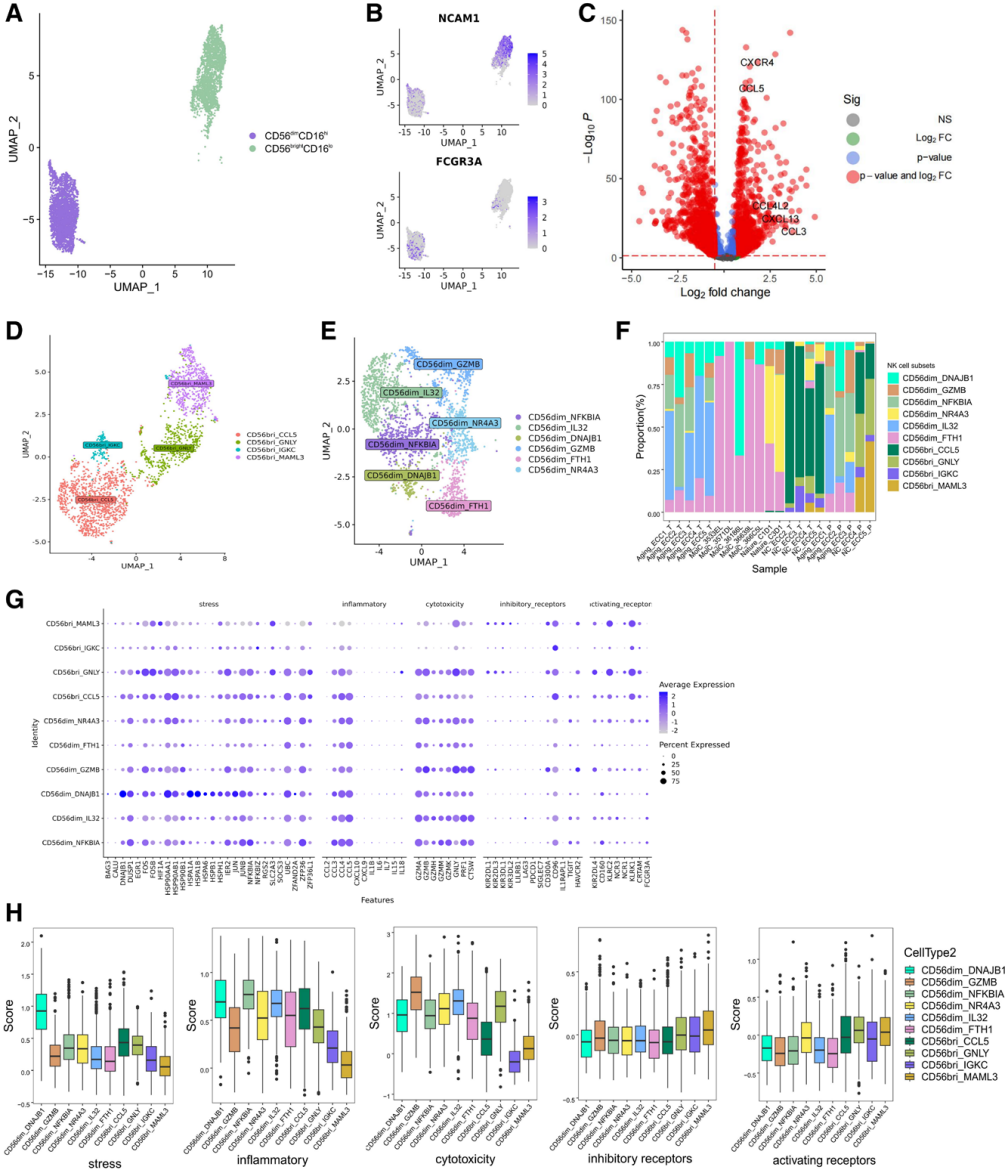
Cellular composition of NK subsets and CD56dim_DNAJB1 dysfunctional state. (A) UMAP plot of NK cells, colored by 2 subsets.CD56^bright^CD16^lo^. (B) Feature plot show log-normalized expression of markers of NK subsets. (C) Volcano plot shows different expression genes of subset between Tumor and Normal groups. (D) UMAP plot of CD56^bright^CD16^lo^ cells, colored by their 4 subsets. (E) UMAP plot of CD56^dim^CD16^hi^ cells, colored by their 6 subsets. (F) Ten NK subsets composition of each patient. (G) Box plot shows the different scores among 5 gene sets, including stress, inflammatory, cytotoxicity, inhibitory receptors and activating receptors.

We next sought to elucidate the specific characteristics of NK cells in the TME of ECC. To accurately identify NK subtypes, we conducted 2 rounds of unsupervised clustering. The first round of analysis focused on differentiating between 2 well-known cell types: CD56^bright^CD16^lo^ (n = 2222) and CD56^dim^CD16^hi^ (n = 2619) (Fig. 3A). This differentiation was based on the expression levels of NCAM1 (CD56) and FCGR3A (CD16) (Fig. 3B). The CD56^dim^CD16^hi^ NK cell population is primarily responsible for killing target cells through the secretion of perforin and granzymes, while the CD56^bright^CD16^lo^ NK cell population exhibits immunoregulatory and cytokine-producing capabilities.^[26,27]^ As expected, our findings revealed that CD56^dim^CD16^hi^ NK cells had higher expression of cytotoxic effector genes, such as PRF1, GZMB, GZMA, GZMH, and GZMK. On the other hand, CD56^bright^CD16^lo^ NK cells showed higher expression of XCL1, XCL2, and CCL4L2. Interestingly, our analysis did not capture the CD56^bright^CD16^hi^ NK cell subtype, which is characterized by high levels of NCAM1 and FCGR3A. This particular NK subtype has been observed in various cancer types and been sought to represent a temporary maturation stage. Furthermore, we did not identify clusters that specifically expressed high levels of proliferation markers like MKI67 and TOP2A, commonly found in many other cancers.^[28]^ These findings highlighted the heterogeneity among different cancer types.

In comparison to the adjacent non-tumor tissues, the CD56^dim^CD16^hi^ NK cells within tumors showed a decrease in the expression of cytotoxic effector genes, such as PRF1 and most granzymes (GZMA, GZMH, and GZMM), except for GZMB. This indicates a potential impairment in their ability to kill target cells. Additionally, these CD56^dim^CD16^hi^ NK cells in the tumor exhibited higher levels of NR4A1 (Table S3, Supplemental Digital Content, http://links.lww.com/MD/L969), a known mediator of T cell dysfunction, which is believed to limit the effectiveness of CAR T cells in solid tumors.^[29]^ Interestingly, our findings also revealed that, the CD56^bright^CD16^lo^ NK cells within tumors showed increased production of cytokines. This was demonstrated by the elevated expression of CCL3, CCL4, CCL5, and XCL2, with the exception of XCL1 (Fig. 3C; Table S4, Supplemental Digital Content, http://links.lww.com/MD/L970). This observation contradicted what is typically observed in other types of solid cancers. Overall, these results suggested a significant change in the functionality of NK cells within ECC TME.

During the second-round clustering, the CD56^bright^CD16^lo^ compartment was divided into 4 subsets (Fig. 3D). Among these subsets, the CD56bri_CCL5 subset was characterized by the specific expression of certain cytokines, including CCL5, CXCR4, CCL4L2, CCL4, CCL3L1, and CCL3. On the other hand, the CD56bri_GNLY subset exhibited a distinct expression pattern, with specific expression of genes associated with cytotoxicity, such as GNLY, NKG7, GZMB, GZMA, and CTSW. For CD56^dim^CD16^hi^ NK cells, 6 subsets were further identified (Fig. 3E). The CD56dim_DNAJB1 subset specifically expressed genes related to stress response, including DNAJB1, HSPA1A, and HSP90AA1. Additionally, this subset showed high levels of NR4A1, which has been recognized as a key mediator of T cell dysfunction. Therefore, this CD56dim_DNAJB1 subset corresponds to the recently reported TaNK population.^[28]^ Notably, the CD56dim_NFKBIA subset also exhibited specific expression of certain cytokines, such as CCL3, CCL4, and CCL4L2, suggesting their ability to recruit other immune cells. The CD56dim_GZMB subtype showed significantly high expression of GZMB and GZMH, indicating its cytotoxic capability. The CD56dim_IL32 subtype exhibited upregulation of IL32, while the CD56dim_NR4A3 subset was characterized by an upregulation of KLRK1 (Killer Cell Lectin Like Receptor K1) and B3GAT1 (CD57). Additionally, CD56dim_GZMB, CD56dim_IL32 and CD56dim_NR4A3 subsets demonstrated high expression of KLRC2 (NKG2C), which is typically associated with adaptive NK cells. The distribution of NK cell subsets was evaluated across samples, revealing significant differences among them (Fig. 3F). Specifically, the CD56dim_DNAJB1 subset was predominantly found in the MolC_36186L patient, while it was not present in MolC_36639L. These findings highlight the heterogeneity in the distribution of NK subsets among patients, which could potentially impact their immune response (Fig. 3G).

We further analyzed the gene expression signature to investigate the functional differences between different NK subsets Notably, compared to all NK cell subsets, the CD56dim_DNAJB1 subset exhibited relatively high expression levels of genes associated with stress (Fig. 3G), resulting in the highest stress score (Fig. 3H). Furthermore, the CD56dim_DNAJB1 subset also displayed a relatively high inflammatory score. Additionally, among the 6 CD56^dim^CD16^hi^ subsets, the CD56dim_DNAJB1 subset exhibited the weakest cytotoxic capability. This suggests a unique role for the CD56dim_DNAJB1 subset in stress response and inflammation, potentially influencing the overall functionality of the NK cell population. To characterize the activation state of NK cells, we assessed the expression levels of inhibitory and activating receptors, both HLA-dependent and HLA-independent, on the surface of NK cells. Intriguingly, we observed no significant variation in the expression of these receptors across the ten NK cell subsets. These findings were in contrast to a recent study, where distinct variations were reported among different NK cell subsets.^[28]^ Overall, our study provides a detailed transcriptome profile of NK cells and reveals significant functional differences among subsets. Furthermore, we identified previously unrecognized heterogeneities between ECC and other cancer types.

### 3.5. Detailed analysis of myeloid cells uncovered LAMP3+ DC impaired activation

**Figure 4.**
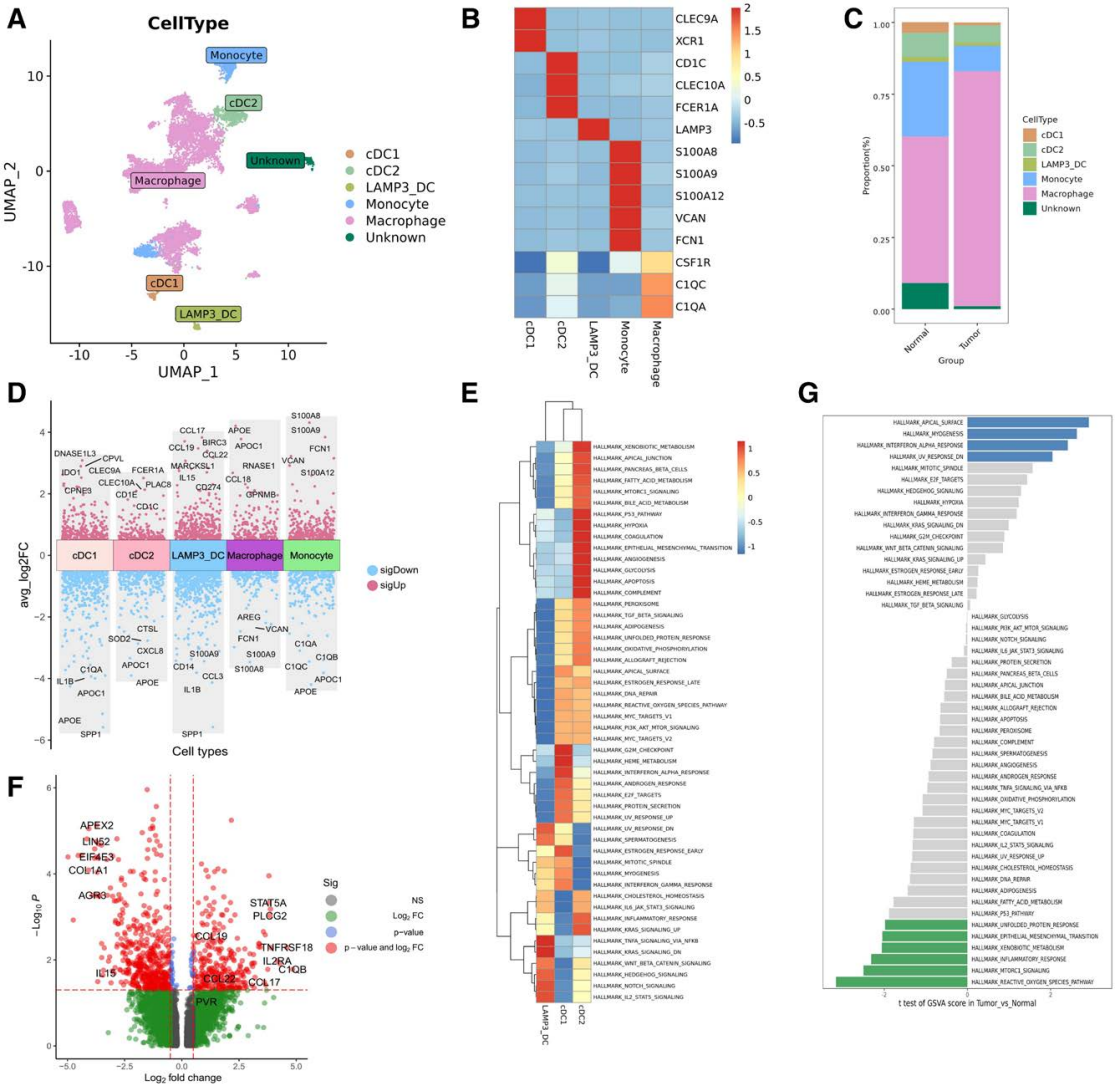
Cellular composition of myeloid cell types. (A) UMAP plot of myeloid cells, colored by 5 cell types and one unknown cluster. (B) Heatmap shows the expression levels of markers of 5 cell types. (C) Myeloid cell type composition of tumor/normal group. (D) Different expressed genes among 5 cell types. Significantly downregulated and upregulated genes were labeled with blue and red, respectively. (E) Heatmap shows difference in pathway activities scored by GSVA per cell among 3 DC cell types. (F) Volcano plot shows different expression genes of LAMP3+ DC cells between tumor and normal groups. (G) Significant different enriched pathways of LAMP3+ DC cells between tumor and normal groups. Significantly upregulated and downregulated pathways in tumor were labeled with blue and green, respectively.

Reclustering of myeloid cells identified 5 distinct cell types: tumor-associated macrophages (C1QA+ C1QC), monocytes (S100A8+ S100A9+), cCD1 cells (classical dendritic cells [DCs], CLEC9A+ XCR1+), cCD2 cells (CD1C+ CLEC10A+) and LAMP3+ DCs (LAMP3+ CCR7+) (Fig. 4A and B). It was worth noting that one cluster displayed markers of both myeloid and T cells, indicating the presence of doublets. Additionally, the analysis revealed that monocytes were predominantly derived from normal tissues, while tumor-associated macrophages were more abundant in tumor tissues, although not significantly (Fig. 4C).

Among the subsets of DCs, LAMP3+ DCs have recently been identified as a mature form of conventional DCs, also known as mregDC.^[30,31]^ In this study, we observed a total of 146 LAMP3+ DCs, with 124 cells found in tumor tissues and 22 in normal tissues. Previous studies have reported that LAMP3+ DCs can attract Treg cells to the tumor area through cytokines.^[31]^ Specifically, CCL17 and CCL22, which bind to CCR4 on the Treg cell membrane, have strong chemotactic activity towards Tregs.^[32,33]^ Interestingly, LAMP3+ DCs in ECC predominantly expressed genes encoding cytokines such as CCL17, CCL19, and CCL22 (Fig. 4D). Additionally, LAMP3+ DCs in tumor tissues showed higher levels of CD274 compared to those in normal tissues (Fig. 4D), suggesting that LAMP3+ DCs infiltrating the tumor region could directly inhibit CD8+ T cells or attract Tregs to the tumor area. Compared to cDC1 and cDC2 subsets, LAMP3+ DCs were significantly enriched in TNFA, KRAS, HEDGEHOG, NORCH, and IL2_STAT5 signaling pathways (Fig. 4E).

Recent immunofluorescence analyses have revealed that LAMP3+ DCs co-localize with NK cells in various types of cancer.^[28]^ The interaction between LAMP3+ DCs and CD56^dim^CD16^hi^ NK cells through the IL-15-IL-15 receptor and NECTIN2-TIGIT interaction axes has been confirmed by CellPhoneDB analysis.^[28]^ This study found that LAMP3+ DCs express the highest levels of IL15 and NECTIN2 among myeloid cells (Fig. 4D). IL-15 is a cytokine crucial for the maintenance of NK cell longevity and homeostasis. Further investigation into the regulatory process of LAMP3+ DCs in tumors revealed that tumor-infiltrating LAMP3+ DCs have lower expression of IL15 compared to those in adjacent non-tumor tissue. These results suggested that LAMP3+ DCs may have impaired activation effects on CD56^dim^CD16^hi^ NK cells in the TME (Fig. 4F). Moreover, compared to LAMP3+ DCs in normal tissues, the tumor-infiltrating LAMP3+ DCs showed significant upregulation in HALLMARK APICAL SURGACE, HALLMARK MYOGENESIS, and HALLMARK INTERFERON ALPHA RESPINSE pathways (Fig. 4G).

### 3.6. Cell–cell communication networks between NK and other immune cells

**Figure 5.**
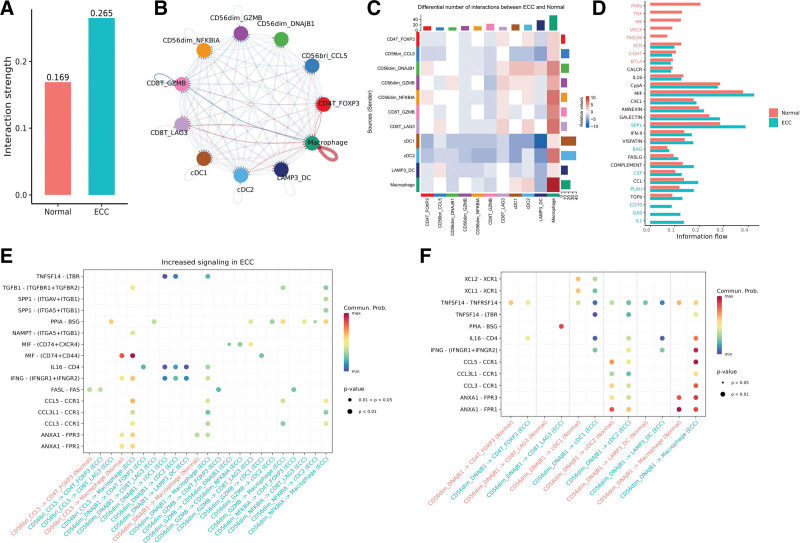
Cell–cell communication networks among 11 cell types. (A) The interaction strength of 11 cell types in normal and tumor. (B) Compared with normal group, the increased (red line) and decreased (blue line) interactions in Tumor group. (C) Heatmap of differential interactions of clusters in the cell–cell communication network. The top bar indicates the sum of incoming signaling, and the right bar indicates the sum of outgoing signaling. (D) All the signaling pathways of Normal and Tumor samples are presented in overall information fow. (E) Compared with Normal group, the increased signalings in tumor group. (F) Dot plot shows the different communication probability in CD56dim_DNAJB1 and CD56bri_CCL5 with other cell types.

To investigate the global communications among cell types and identify ECC-specific signaling pathways, we conducted a combined analysis of ECC and normal (normal) datasets using CellChat. These datasets included 11 cell types for both ECC and normal, consisting of 4 myeloid cell types (cDC1, cDC2, LAMP3_DC, macrophage), 3 T subsets (CD8T_GZMB, CD8T_LAG3, CD4T_FOXP3), 3 CD56^dim^CD16^hi^ NK subsets (CD56dim_DNAJB1, CD56dim_GZMB, CD56dim_NFKBIA), as well as the CD56^bright^CD16^hi^ subset CD56bri_CCL5. Firstly, we calculated the interactions for each dataset separately, resulting in the detection of 23 and 25 signaling pathways in ECC and normal, respectively. Subsequently, we compared the total number of interactions and the strength of cell–cell communication networks. Our findings revealed that cell–cell communication was enhanced in ECC compared to normal (Fig. 5A), particularly between macrophages, CD56dim_DNAJB1, and DCs cells (Fig. 5B).

Our findings revealed that cDC1 and cDC2 cells, acting as the primary senders, exhibited the strongest outgoing interaction strength. These interactions were found to be diminished in ECC. On the other hand, CD56dim_DNAJB1 cells also demonstrated a relatively high outgoing interaction strength, primarily interacting with macrophage and DCs. Notably, these interactions were more pronounced in ECC compared to normal cells (Fig. 5C). In addition, we analyzed the information flow of each signaling pathway. We found that CALCR, IL6, and MIF pathways exhibited a consistent flow, implying their comparable significance in both ECC and normal cells (Fig. 5D). On the other hand, we observed upregulation of SPP1, BAG, and CSF pathways in ECC patients, indicating their potential involvement in the disease. Moreover, 3 pathways, namely IL1, GAS, and CD70, were exclusively active in ECC, suggesting their crucial roles in the progression of ECC (Fig. 5D).

When compared with normal group, CD56bri_CCL5 cells in tumor demonstrated an increased interaction weight with macrophage cells through the MIF-(CD74+ CD44) ligand-receptor pair (Fig. 5E). MIF, as known as macrophage migration inhibitory factor, plays a role in regulating macrophage function by suppressing anti-inflammatory effects. CD74 has been reported to play a crucial role in maintaining tumor homeostasis by releasing a signal that inhibits T cell activity, thereby facilitating tumor escape.^[34]^ Additionally, CD44 variants may contribute to the epithelial–mesenchymal transition and adaptive plasticity of cancer cells.^[35]^ We also observed a significant increase in interactions between CD56dim_DNAJB1 and macrophage cells through several ligand-receptor pairs, such as CCL3-CCR1, CCL3L1-CCR1, CCL5-CCR1, IFNG-(IFNGR1+ IFNGR2), IL16-CD4, and TNFSF14-LTBR (Fig. 5F).

## 4. Discussion

In this study, we have collected a large dataset of scRNA datasets of ECC, consisting of 16 ECC and 6 adjacent normal tissues. As well as we known, this dataset is currently the largest available, which providing a strong foundation for our comprehensive analysis. Our study primarily focused on tumor-infiltrating NK cells, and we investigated the CD56brightCD16lo and CD56dimCD16hi subsets exhibited functional diversity. Additionally, we uncovered the complex interactions between NK and other immune cell types in the TME. Through this comprehensive analysis, our study provides valuable insights into the role and dynamics of NK cells within the ECC TME.

NK cells play a pivotal role in tumor control, participating in various processes. Enhancing the infiltration of NK cells into solid malignancies has been a central objective in the development of therapeutic NK cell products.^[36]^ Furthermore, the decrease in tumor-infiltrating NK cells might represent a potential mechanism by which tumors evade NK cell immunosurveillance.^[37–39]^ In our study, we observed a significant reduction in the proportion of NK cells in tumor tissues compared to normal tissues, a finding that aligns with observations in other cancer types. We also observed potential dysfunction in tumor-infiltrating NK cells. The CD56dimCD16hi subsets of NK cells within the tumor tissue showed lower expression levels of cytotoxic genes like PRF1, GZMB, GZMA, GZMH, and GZMK compared to normal tissues. Both the CD56dimCD16hi and CD56brightCD16lo subsets exhibited low expression of B3GAT1 (CD57) and the killer immunoglobulin-like receptor family, indicating immaturity and reduced killing capacity. This dysfunction of NK cells, similar to T cell exhaustion, suggested impaired natural cytotoxicity in ECC. It has been reported that breast cancer patients who responded positively to monotherapy with trastuzumab (an anti-HER2 antibody) exhibited significantly higher levels of both NK and antibody-dependent cellular cytotoxicity activities compared to nonresponders.^[40]^ This suggests a possible correlation between therapy response and the level of these immune activities. Additionally, this finding indicates that the dysfunctional state of tumor-infiltrating NK cells in ECC may potentially impact the effectiveness of antibody-based drugs through the antibody-dependent cellular cytotoxicity mechanism. Therefore, gaining a comprehensive understanding of and addressing the dysfunction of NK cells could be crucial in improving therapeutic outcomes for ECC patients.

CAR-T cell immunotherapies have achieved indisputable clinical successes in several recalcitrant cancer types. However, the number of patients achieving durable responses remains relatively limited.^[4]^ Unlike CAR-T immunotherapies, which can have toxic side effects, CAR-NK cells are capable of simultaneously improving efficacy and controlling adverse effects, such as acute cytokine release syndrome, neurotoxicity, and graft-versus-host disease.^[5]^ Meanwhile, CAR-NK cell therapies have also achieved remarkable clinical success in treating hematological malignancies, such as lymphoma, myeloma, and leukemia.^[5,41]^ However, the effectiveness of CAR-NK therapies in solid tumors is partly hindered due to the heterogeneity and impaired state within the TME. The dysfunctional state or exhaustion of CD8 + T cells within the TME, a key reason for CAR-T nonresponse, is relatively well-studied. Several inhibitory receptors, such as PD-1, CTLA4, TIGIT, and TIM3, have been identified. However, it is still unclear whether these immune checkpoints play the same role in the dysfunction of NK cells. In this study, we identified a subset of NK cells, CD56dim_DNAJB1, akin to the TaNK cells recently discovered by Tang et al.^[28]^ TaNK cells have been associated with poor prognosis and resistance to immunotherapy in various cancer types. Although the CD56dim_DNAJB1 subset was not the predominant tumor-infiltrating NK cells in ECC, we hypothesize that this subset may influence or reflect the tumor immune responses in ECC. Firstly, the CD56dim_DNAJB1 subset demonstrated the weakest cytotoxic capability and showed a relatively high inflammatory score. Secondly, this subset exhibited a significant stress state, suggesting its involvement in the tumor immune response. Conventional immune checkpoint genes such as PDCD1 and CTLA4 were minimally expressed on CD56dim_DNAJB1 cells, indicating that they may not be direct targets of anti-PD-1/CTLA-4 therapies.^[42,43]^ Therefore, CD56dim_DNAJB1 cells may have distinct roles from Tex cells in the TME and the current ICB treatments.

Meanwhile, we observed that NK subsets had strong cell–cell communication networks with other cell populations in the TME. CD56bri_CCL5 cells showed increased interaction with macrophage cells through the MIF-(CD74 + CD44) ligand-receptor pair (Fig. 5E). We also noted a significant increase in interactions between CD56dim_DNAJB1 and macrophage cells through several ligand-receptor pairs (Fig. 5F). These results collectively suggested tumor-infiltrating NK cells in ECC are sensitive to immunosuppressive factors of the TME, which may contribute to their dysfunctional phenotype.

In summary, our comprehensive analyses enhance the current understanding of TME of ECC. We also revealed the NK cells dysfunctional states and their strong communications with other immunosuppressive factors. These findings have the potential to guide the development of more effective NK-cell based immunotherapeutic strategies for ECC. In addition, our results illuminate insights into CAR-NK immunotherapies in ECC and other solid tumors.

## Acknowledgments

We thank all the participants in this study. This work was supported by Prof. Wang Yuping from Xuanwu Hospital Capital Medical University.

## Author contributions

**Conceptualization:** Wenjie Shi, Jing Wang, Xianghong Meng.

**Data curation:** Wenjie Shi, Wuchen Wu, Jing Wang.

**Formal analysis:** Wuchen Wu.

**Funding acquisition:** Xianghong Meng.

**Investigation:** Wenjie Shi.

**Methodology:** Wenjie Shi, Wuchen Wu.

**Software:** Jing Wang.

**Supervision:** Jing Wang, Xianghong Meng.

**Validation:** Jing Wang.

**Writing – original draft:** Wenjie Shi, Xianghong Meng.

**Writing – review & editing:** Wenjie Shi, Xianghong Meng.

## Supplementary Material








